# Darwin’s Green Living Fossil: The Microalga *Cyanophora paradoxa* and Evolutionary Stasis

**DOI:** 10.3390/microorganisms12081511

**Published:** 2024-07-23

**Authors:** Ulrich Kutschera, Rajnish Khanna

**Affiliations:** 1 I-Cultiver, Inc., 6102 Shoshone Dr, Manteca, CA 95336, USA; 2 Department of Plant Biology, Carnegie Institution for Science, 260 Panama St., Stanford, CA 94305, USA

In 1723, Anthonie van Leeuwenhoek, the “master of fleas and father of microbiology”, died at the age of 90 [1]. Two centuries later, the Russian biologist A. Korschikov described the freshwater alga *Cyanophora paradoxa* n. sp. n. gen. (Glaucophyta) based on individuals collected in a pond in Charkow, Ukraine [2]. *C. paradoxa* was interpreted as a “Living Fossil”, because the cell contains “cyanelles”, i.e., archaic plastids surrounded by a prokaryotic peptidoglycan wall. Recently, this view has been questioned (see ref. [3]).

In this Editorial, we present an English translation of this classical paper and novel data on *C. paradoxa* Korschikov 1924 and summarize recent findings regarding the structure and function of photosystem I in this microbe and others. These new data document that *C. paradoxa* should be classified as “Darwin’s Green Living Fossil”, because this unique flagellate fulfills all the criteria for such an ancient–extant aquatic microalga.

Over the past few decades, we have studied the developmental biology and physiology of aquatic cyanobacteria (*Anabaena* sp.) and green algae (*Clamydomonas* sp., *Chlorella* sp., *Coleochaete* sp., *Mesostigma* sp., inclusive of the glaucophyte *Cyanophora paradoxa*) maintained in liquid media, as well as bryophytes, ferns and seed plants [4].

Accordingly, we read the literature on *C. paradoxa* with great interest. However, we noted that, in these sophisticated papers, the original description of this “Living Fossil” microbe has been ignored (see, for instance, refs. [3,5,6]). This may be due to the fact that Korschikov (1924) [2] published his seminal article in an obscure journal (*Russij Archiv Protistologii*) in Russian, with an Abstract in German. The content of this key paper [2], published one century ago, can be summarized as follows.

In his article entitled “Protistologische Beobachtungen” (“Observations on Protists”), the following four new freshwater species are described:

1. *Cyanophora paradoxa*, n. g. n. sp.;
2. *Cryptomonas lobate* n. sp.;
3. *Ochromonas pallida* n. sp.;
4. *Characium ocellatum* n. sp.

Here, we will focus on *C. paradoxa*, a new genus and new species. Figure 1A shows the original drawings from the article by Korschikov, 1924 [2]. The author characterized his nova species (and new genus) in the following words (translated into English):

A typical individual of *C. paradoxa* is 6 to 9 micro-meters long and is equipped with 2 flagella, one of which is much shorter than the other; the only contractile vacuole is in the rear part, and the nucleus is in the front region of the cell. In the center of the microbe, two large green bodies are visible, which contain a colorless, dense granum. Starch grains are detectable in the cytoplasm, often in the vicinity of the nucleus. The green bodies divide, reminiscent of microbes of the genus *Chroococcus*.

*C. paradoxa* reproduces via longitudinal cell division, usually when the aquatic organism swims around with the aid of its motile flagella. The green bodies are equally distributed among the dividing cells. Sometimes, encystation is observed: the protoplast becomes round, the nucleus moves to the center of the cell and the contractile vacuole disappears. Starch is stored in large granules; a thin wall surrounds the rest of the cell.

The colored bodies should not be interpreted as chromatophores (chloroplasts); rather, they represent independent organisms. One can squeeze them out of the host organism, and thereafter they remain, cultivated in aqueous solutions, intact over long time periods. Hence, the green bodies are independent organisms, which live in symbiosis with the colorless flagellate. The nature of these symbionts remains elusive. However, they are reminiscent to cyanophyceen (i.e., Cyanobacteria), but concerning their structure, they differ from typical blue-green algae. In summary, *C. paradoxa* must be viewed as an organism sui generis, i.e., a unique one of a kind, with no analogy to any other living being known so far.

Locus typicus: Region Charkow; occurs in small muddy waters, but never at high density.

Therefore, according to Korschikov (1924), the “Green pond microbes from Charkow” represent a symbiotic system, consisting of a flagellate (host) and “green bodies reminiscent of cyanobacteria” (symbionts) [2].

Decades later, Steiner and Löffelhardt [5] and other biologists interpreted *C. paradoxa* (Figure 1A–C), the best-studied species within the glaucophyte algae (Glaucophyta, one branch of the Archaeplastida, i.e., Glaucophyta, Rhodophyta, Viridiplantae), as a “Living Fossil”.

These authors argued that the two “green intracellular bodies” are the “plastids” of *C. paradoxa*, because ”the transition from endosymbiont to organelle has certainly taken place”, [5]. In order to emphasize the plastid nature of the ”cyanelle”, alternative names have been coined, such as “cyanoplast” or “muroplast”. The third term signifies that the “cyanelle” is surrounded by a prokaryote-like peptidoglycan wall. This “Living Fossil” may “mimic an early stage in organelle evolution” and, hence, is strong proof of the “endosymbiotic theory”, describing and explaining the origin of plastids in green eukaryotes (algae, plants, i.e., the Viridiplantae; see [7,8]).

Ten years after Steiner and Löffelhardt’s account, Price et al. [3] published the draft nuclear genome and part of the proteome of *C. paradoxa* (the sequence of the cyanelle genome, which is of similar size to that of typical chloroplasts, has been known since 1995). Based on these data, there is strong evidence for a single evolutionary origin of the primary plastids in the “Supergroup Plantae”. In addition, the hypothesis emerged that the glaucophytes may not be a lineage which should be interpreted as “Living Fossils”. The reasons for this surprising statement are as follows: In addition to the “primitive” peptidoglycan wall that surrounds the cyanelles, *C. paradoxa* has several derived traits, such as a modern pathway for starch biosynthesis and sophisticated plastid protein-import machinery. Nevertheless, the nuclear genome of *C. paradoxa* contains a signal of cyanobacterial ancestry.

Moreover, some key genes responsible for starch biosynthesis were found to be derived from prokaryotic “energy parasites”, such as Chlamydiae. Price et al. [3] concluded that the *C. paradoxa* genome contains a unique combination of ancestral, novel, and borrowed (via horizontal gene-transfer-acquired) genes, like the genomes of members of the Kingdom Plantae.

In the most recent analysis of an improved *C. paradoxa* genome assembly, Price et al. [6] corroborate their earlier findings and conclude that this “paradox microorganism” is an important “model for understanding ancient events in algal evolution”. Moreover, they speculate that “additional genomes of glaucophyte species (should be analyzed) to discover…ancient events driven by endosymbiosis in the ancestor of these taxa” [6].

In this context, studies on the cyanobacterium *Anabaena* sp. and *C. paradoxa* by Kato et al. [9,10], which analyzed the structure of photosystem I in these microorganisms, are of importance.

During oxygenic photosynthesis, solar energy is converted into chemical energy (stored, for instance, as starch grains). In addition, molecular oxygen (O_2_) is generated via the light-mediated splitting of water (H_2_O). In all green oxygenic photosynthesizers, this light-driven energy conversion is carried out by two pigment–protein complexes (consisting of several subunits)—photosystem II (PS II, usually organized into a dimer) and PS I (diverse among photoautotrophic organisms) [11].

For instance, in the well-studied cyanobacterium *Anabaena* sp., the structure of the PSI tetramer, as well as the excitation energy transfer processes, upon photon absorbance, have been characterized in detail [9]. In a subsequent study, Kato et al. [10] documented that the PSI tetramer isolated from the cyanelle of *C. paradoxa* displays a special monomer–monomer arrangement (and interaction) that differs considerably from that observed in the *Anabaena* tetramer [10]. Moreover, the excitation energy transfer patterns in *C. paradoxa* PS I tetramers were found to be different from that in the cyanobacterium analyzed by the authors.

Based on these comparative studies (PS I in *Anabaena* sp. vs. *C. paradoxa*), Kato et al. [10] concluded that the “Cyanophora PS I represents an evolutionary turning-point between cyanobacteria and other photosynthetic eukaryotes” (such as algae and land plants).

In another section of their research paper, Kato et al. [10] argue that “*Cyanophora* is an intermediate between oxygenic photosynthetic prokaryotes and eukaryotes in the evolutionary processes of oxyphototrophs”. More specifically, the authors drew the following far-reaching conclusion: “*Cyanophora* is evolved from cyanobacteria having PS I-trimers and tetramers, and subsequently serves as an ancestor for other eukaryotic algae where PS I becomes a monomer”, and “Cyanophora PS I is in the middle of transition from cyanobacterial trimers and tetramers to eukaryotic monomers” [10]. These statements raise the following question:

How are “Living Fossils” defined?

In his book *On the Origin of Species*, Darwin (1859) [12] wrote that “aberrant species”, which “may fancifully be called ‘living fossils’,… will aid us in forming a picture of the ancient forms of life”. As examples, Darwin [12] mentioned the “Ornithorhynchus” (Platypus) and the South American lungfish *Lepidosiren* as “Living Fossils”, because they may “connect to a certain extent orders now widely separated in the natural scale” [12].

As detailed by Rieppel [13], today, “Living Fossils” are interpreted as extant organisms that “have retained a few prominent primitive traits”… and … “that have not changed over long periods of geological time”. Nevertheless, “Living Fossils” also have evolved specializations, such as the exoskeleton of the pectoral fin in the “primitive” Coelacanth (*Latimeria* sp.). Recently, Brownstein et al. [14] have shown that two ancient “Living Fossils”—groups of ray-finned fish, gars (*Atractosteus* sp.) and sturgeons (*Acipenser* sp.)—display the lowest rates of molecular evolution among 481 vertebrate species. Hence, “molecular stasis” (i.e., a low rate of nucleotide substitution in protein-coding genes) may be one mechanism for slow phenotypic changes in key traits of these ancient organisms [14].

Here, we have documented that the “100-year-old microalga *C. paradoxa* Korschikow 1924” [2] qualifies as a paradigmatic “Green Living Fossil” based on two “primitive” features: the bacteria-like peptidoglycan wall that surrounds the two cyanelles and the intermediate “connecting link-like” structure and function of photosystem I within the “muroplasts” of these photosynthetically active flagellates. However, more work is required to further corroborate the conclusion that Korschikow’s “paradox pond microbe” is a symbiotic system consisting of very ancient and “modern” traits and to elucidate whether the principle of “molecular stasis” [14] applies to the microalga discussed here (Figure 1A–C).

## Figures and Tables

**Figure 1:**
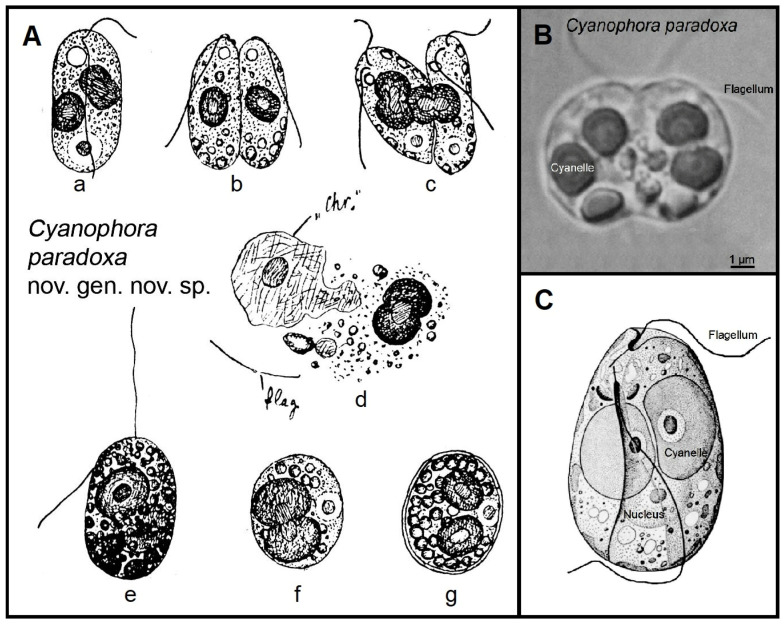
Original drawings by A. Korschikov, 1924, depicting the aquatic microalga *Cyanophora paradoxa* (**A**). Adult individuals in the process of cell division (**a**–**c**) and a destroyed microorganism, showing two chromatophores (**d**). Fixed individual (**e**), start of encystment (**f**) and a fully developed cyst (**g**) (adapted from ref. [2]). Light micrograph of an adult, living, dividing individual of *Cyanophora paradoxa* (**B**) and a schematic drawing of this unicellular microalga (**C**).

## Data Availability

Not applicable.
